# Genome-Wide Single-Nucleotide Polymorphism-Based Genomic Diversity and Runs of Homozygosity for Selection Signatures in Equine Breeds

**DOI:** 10.3390/genes14081623

**Published:** 2023-08-14

**Authors:** Anuradha Bhardwaj, Gitanjali Tandon, Yash Pal, Nitesh Kumar Sharma, Varij Nayan, Sonali Soni, Mir Asif Iquebal, Sarika Jaiswal, Ram Avatar Legha, Thirumala Rao Talluri, Tarun Kumar Bhattacharya, Dinesh Kumar, Anil Rai, B. N. Tripathi

**Affiliations:** 1 ICAR-National Research Centre on Equines, Sirsa Road, Hisar 125001, India; yashpal1888@gmail.com (Y.P.);; 2 Division of Agricultural Bioinformatics, ICAR-Indian Agricultural Statistics Research Institute, New Delhi 110012, India; gitanjali.iasri@gmail.com (G.T.); sharmanitesh.iasri@gmail.com (N.K.S.); dinesh.kumar@icar.gov.in (D.K.);; 3 ICAR-Central Institute for Research on Buffaloes, Hisar 125001, India; varij.nayan@icar.gov.in; 4 Indian Council of Agricultural Research, Krishi Bhawan, New Delhi 110001, India

**Keywords:** *Equus caballus*, runs of homozygosity (ROH), SNP, QTL

## Abstract

The horse, one of the most domesticated animals, has been used for several purposes, like transportation, hunting, in sport, or for agriculture-related works. Kathiawari, Marwari, Manipuri, Zanskari, Bhutia, Spiti, and Thoroughbred are the main breeds of horses, particularly due to their agroclimatic adaptation and role in any kind of strong physical activity, and these characteristics are majorly governed by genetic factors. The genetic diversity and phylogenetic relationship of these Indian equine breeds using microsatellite markers have been reported, but further studies exploring the SNP diversity and runs of homozygosity revealing the selection signature of breeds are still warranted. In our study, the identification of genes that play a vital role in muscle development is performed through SNP detection via the whole-genome sequencing approach. A total of 96 samples, categorized under seven breeds, and 620,721 SNPs were considered to ascertain the ROH patterns amongst all the seven breeds. Over 5444 ROH islands were mined, and the maximum number of ROHs was found to be present in Zanskari, while Thoroughbred was confined to the lowest number of ROHs. Gene enrichment of these ROH islands produced 6757 functional genes, with AGPAT1, CLEC4, and CFAP20 as important gene families. However, QTL annotation revealed that the maximum QTLs were associated with Wither’s height trait ontology that falls under the growth trait in all seven breeds. An Equine SNP marker database (EqSNPDb) was developed to catalogue ROHs for all these equine breeds for the flexible and easy chromosome-wise retrieval of ROH along with the genotype details of all the SNPs. Such a study can reveal breed divergence in different climatic and ecological conditions.

## 1. Introduction

The horse, scientifically known as *Equus ferus caballus*, was one of the first domesticated animals and has been used for scores of purposes in human societies. It is used as a companion animal, as well as for food, transportation, draught and agricultural work, and is used in sport, hunting, and warfare [1,2]. In India, there are different species of the family Equidae used for diverse purposes due to the country’s complex arrangement of varying terrains and climatic conditions. The National Commission on Agriculture has identified seven different breeds of horses in India, based on their geographical localization and phenotypic characteristics: Kathiawari, Marwari, Manipuri, Zanskari, Bhutia, Spiti, and Thoroughbred. These breeds of horses differ not just due to their agroclimatic adaptation but also due to their particular performance characteristics [3,4,5,6].

The phenotypical characteristics of Indian equines are highly distinctive and unique in comparison to horses in the rest of the world, which suggests their geographical distribution and subsequent acquisition of selective characteristics over time. The ponies, Spiti and Zanskari, belong to the Himalayan region, making them susceptible to cold temperatures and scarce food availability. Both breeds have high stamina since they can move on steep terrains. Bhutia and Manipuri ponies are similarly capable, with high endurance. Marwari and Kathiawari breeds are understood to be similar to each other due to their close resemblance, as they are related to Arabian horses. Still, Marwari horses are longer than Kathiawari. Kathiawari horses are considered to be the offspring of wild horses of Gujarat and Arabian Sea [4].

A variety of genomic tools have been used to study the genome and functions of modern equines. Considering equines across the world, several generations of high-throughput genotyping arrays known as “single nucleotide polymorphism (SNP) chips” have been released since the equine genome was first sequenced, enabling rapid and cost-effective genotyping. A genotyping array with 54,602 SNPs (Illumina EquineSNP50 BeadChip; Illumina, San Diego, CA, USA) was developed using SNPs identified via Sanger sequencing of a small group of horses [7]. Additionally, Illumina released a higher-density array (Illumina EqineSNP70 BeadChip) with 65,157 SNPs. SNP50 and SNP70 BeadChips have been superseded by the MNEc670 K array, which uses SNPs identified from whole-genome sequences of 153 horses to survey 670,805 sites across the equine genome [8]. Due to an increase in short-read-based whole-genome sequencing (WGS), coupled with advances in chip-based genotyping technologies, the HGS array MNEc2M and the HGS array MNEc670k were entirely redesigned in February 2017.

One of the most common concerns about equines is their physical deformities, which occur due to their racing- and performance-based activities, as well as genetic factors [9]. A lot of equines suffer from skeletal-related diseases. One such disease is skeletal ativism, which causes abnormal growth of the uvula and tarsal joints, sometimes leading to the death of the horses. A similar study has been conducted on Sweden Shetland ponies and their mutations have been detected, leading to the cause of ativism. Using the method of whole-genome sequencing through SNP detection, the importance and significance of the gene SHOX in the muscle development of horses have been determined [10]. Divergent genetic population studies were also conducted using an approximately 670,000-SNP genotyping array in European horse breeds [11]. The study highlighted the putative target genes like embryonic morphogenesis (HOXB), energy metabolism (IGFBP-1, IGFBP-3), and autophagy (RALB). This study conducted the experiment on 1476 horses belonging to 12 breeds. Population-based studies are important in horses in order to help us understand their genetic variations and domestication possibilities. Equine SNP arrays have been successfully used in the discovery of variation associated with important traits [12,13,14,15,16].

Though a study on the genetic diversity and phylogenetic relationship of Indian equine breeds like Spiti, Zanskari, Manipuri, Bhutia, Kathiawari, and Marwari using microsatellite markers was reported [3,4,6], there are no reports on SNP diversity and runs of homozygosity revealing the selection signature of breeds. Such a study can reveal how breed divergence has occurred in different climatic and ecological conditions.

## 2. Methodology

### 2.1. Samples Collection, DNA Extraction and SNP Genotyping

Blood samples were collected from the jugular vein into a vacutainer with potassium ethylenediaminetetraacetic acid (K2 EDTA) from 07 horse breeds, namely Manipuri (17), Zanskari (20), Bhutia (20), Spiti (16), Kathiawari (10), Marwari (10), and Thoroughbred (3), covering 96 genotypes. All blood samples were transported to the research laboratory in an ice box and stored at 4 °C and processed for DNA isolation.

According to the manufacturer’s instructions, genomic DNA was isolated from blood using a ReliaPrep™ Blood gDNA Miniprep System (Promega, DC, USA). The isolated DNA was stored at −20 °C to prevent DNA degradation. Agarose gel electrophoresis (0.8% agarose gel) and a Qubit4 fluorometer were used to qualitatively and quantitatively analyze DNA (at 260 nm/280 nm absorbance). Samples were genotyped using the Axiom™ Equine Genotyping Array with an average recall rate of 92.6% for each sample.

### 2.2. Data Quality Control and Measurement of Runs of Homozygosity and Their Distribution

For every individual, Runs of Homozygosity (ROH) were computed using PLINK v 1.9 [17,18]. Before estimation of Runs of Homozygosity, a quality check and Linkage Disequilibrium pruning were performed. To check the quality of the SNPs, markers with more than 15% missing values and low-frequency SNPs were eliminated by setting the minor allele frequency (MAF) to less than 5%. Individuals with more than 10% missing SNP calls were removed from the genotype dataset.

To exclude short and common ROH, which were present across the genome, the minimum ROH length was set to 1 Mb so that short ROHs induced by LD effects could be excluded [19]. Other parameters that were set for computing ROH were (i) a sliding window with 50 SNPs across the whole genome; (ii) an overlapping homozygous proportion window at 0.05; (iii) a minimum number of consecutive ROH at 100; (iv) minimum length of ROH at 1 Mb; (v) density of single SNP per 50 kb; (vi) a maximum distance between consecutive homozygous SNPs of 100 kb; and (vii) allowing a maximum of 5 SNPs with missing genotypes and up to one heterozygous genotypes in a ROH. The obtained ROH were congregated into five classes according to the nomenclature of Kirin et al. (2010) and Ferenčaković et al. (2013a; 2013b), i.e., 1–2, 2–4, 4–8, 8–16 and >16 Mb [20,21,22]. For every individual of each breed, the mean number of ROH per individual (M_ROH_), the average length of ROH (A_ROH_) and the total number of ROH per breed (N_ROH_) were computed. The percentage of chromosomes covered by ROH was also computed. To achieve this, the mean ROH length was calculated by adding all the ROHs (in Mb) on each chromosome and dividing by the number of individuals that had ROH on that chromosome. This mean was then further divided by the length of the chromosome (in Mb).

### 2.3. Genomic Inbreeding Coefficients

The genomic inbreeding coefficients (both F_HOM_ and F_ROH)_ were estimated using PLINK v 1.9. F_ROH_ (Inbreeding coefficient) was computed as stated by McQuillan et al. [23], i.e.,

(1) $$F_{ROH}=\frac{\sum L_{ROH}}{L_{AUTO}}$$

where, $\sum L_{ROH}$ refers to the total length of all ROH in an individual genome and $L_{AUTO}$ represents the total autosomal length covered by SNPs included in an array.

Inbreeding based on the observed versus expected number of homozygous genotypes (F_HOM_) was calculated using PLINK v1.90 by computing observed and expected autosomal homozygous genotypes counts for each sample as follows:

(2) $$F_{HOM}=\frac{\mathrm{Number}\mathrm{of}\mathrm{observed}\mathrm{homozygous}\mathrm{loci}-\mathrm{Number}\mathrm{of}\mathrm{expected}\mathrm{homozygous}\mathrm{loci}}{\mathrm{Number}\mathrm{of}\mathrm{nonmissing}\mathrm{loci}-\mathrm{Number}\mathrm{of}\mathrm{expected}\mathrm{homozygous}\mathrm{loci}}$$

### 2.4. Population Diversity Analysis

For population diversity analysis, STRUCTURE v.2.3.1 was used [24], which implements a Bayesian model-based clustering approach that identifies the second-order rate of change of the likelihood (∆K). It estimates the optimal subpopulations and the membership probability of each genotype to the subpopulations. For this analysis, 5 independent iterations for each sub-population were performed under the admixture model of population structure with correlated allele frequencies and 50,000 lengths burn-in period. Then, 100,000 Markov Chain Monte Carlo (MCMC) replications after burn-in were applied for each iteration.

### 2.5. Gene Annotation and Functional Enrichment Analysis

All the SNPs of ROH were annotated to mine the functional genes using SnpEFF [25]. These Genes corresponding to ROH island regions were further used for enrichment analysis. Gene ontology (GO) and Kyoto Encyclopedia of Genes and Genomes (KEGG) pathway enrichment analysis was performed by searching against the GO database using DAVID, a Bioinformatics resource [26]. All horse genes annotated in ENSEMBL were considered the background set. The *p*-value was adjusted using false discovery rates (q values). Subsequently, significance was based on a corrected false discovery rate value set at q ≤ 0.05.

### 2.6. ROH Annotation by Horse QTL Database

Known QTLs of horses, which are falling in ROH regions, were superimposed over the horse QTL map. QTL details were obtained from the Horse QTL database (https://www.animalgenome.org/cgi-bin/QTLdb/EC/index) (accessed on 14 March 2022) for the EC_3.0 assembly and was plotted against top 20 ROH islands. Information used to locate the important traits within the relevant genomic regions was mined and this was carried out based on SNP markers via the filtering criteria.

### 2.7. Development of Equine SNP Marker Database

The Equine SNP marker database (EqSNPDb) was developed using three-tier architecture. It consists of a database layer, an application layer (server), and a presentation layer (client). It is a relational database that provides information about the Runs of Homozygosity (ROH) deciphered from SNPs of the equine breeds under study. All the information about ROHs was saved in the form of tables in MySQL at the database level. For fetching and queries provided by the user, scripting in PHP was conducted in the middle tier, i.e., the application layer. To allow clients to browse the database, web pages were developed in the form of a presentation layer.

## 3. Results

### 3.1. Filtration of Genotypes

The overall genotyping rate for these 96 samples was 99.8%. These 96 genotypes belonged to seven breeds, namely, Manipuri (17), Zanskari (20), Bhutia (20), Spiti (16), Kathiawari (10), Marwari (10) and Thoroughbred (3). A total of 620,721 SNPs were filtered and were carried further for ROH analysis.

### 3.2. ROH Distribution

**Figure 1:**
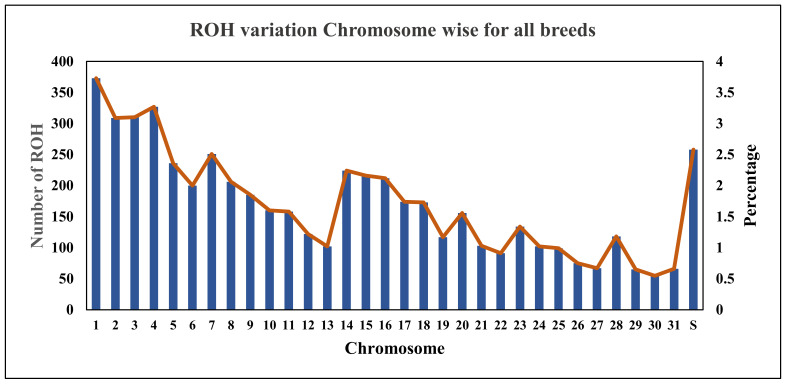
Chromosome-wise ROH variation of autosomes of all equine breeds under study (S = scaffolds). Blue bars represent the chromosome-wise number (frequency) of ROH. Brown line is the frequency curve joining the mid-points of each blue bar.

A total of 5444 homozygous segments were identified. The mean number of ROH was highest for Thoroughbred (1350.84), followed by Kathiawari (1286.31) and Bhutia (1212.11), while the mean number of ROH per animal was highest in Spiti, i.e., 67.69, and lowest in Bhutia, i.e., 48.05. After computing the average length of ROHs for all breeds, it was observed that the Marwari breed had the lowest average length of 4.6 Mb and Thoroughbred had highest average length of 5.7 Mb. A significant number of ROHs were observed on chromosome 1, i.e., 373 (6.85%) and the chromosome 30 had the least number of ROHs, i.e., 66 (1.01%). Details of the ROHs for each chromosome are given in Figure 1. The percentage variation of the ROHs of each chromosome for all breeds is also shown. Breed-wise ROH for each chromosome is given in Supplementary Sheet S1. Chromosome X are the scaffolds showing the number of ROHs.

**Figure 2:**
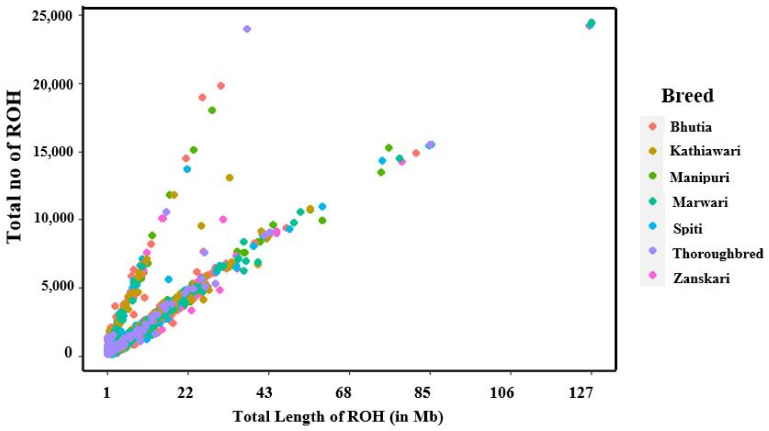
The total number of ROH and length of genome under ROH for each individual in equine breeds.

The total number and length of genomes under ROH for each individual in a breed is given in Figure 2. It was observed that majority of the individuals clustered close to the origin due to the abundant presence of short ROHs in all seven breeds.

**Figure 3:**
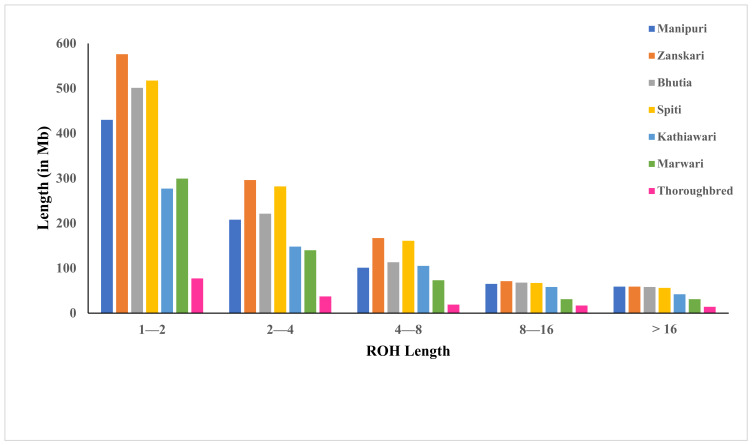
The breed-wise mean sum of ROH. Within each ROH length category, the sum of ROH (in Mb) was calculated per animal and averaged breed-wise.

The proportion of autosomal ROH variation within a breed and between different breeds was studied. When the frequency of ROH categorized according to the length of ROH among all the breeds was plotted, Zanskari showed the maximum length, followed by Spiti and Manipuri (Figure 3).

While studying the percentage of genome coverage by ROH, Manipuri had minimum coverage and Spiti had maximum genome coverage. The average genomic inbreeding (FROH > 1 Mb) coefficient of Spiti was higher and Thoroughbred had the lowest. On the other hand, FROH > 8 Mb of Thoroughbred was the highest and Zanskari had minimum value. Detailed information about the inbreeding coefficient for all the species has been provided in Table 1 above. Statistics of ROH size based on the breeds are provided in Table 2.

### 3.3. Population Diversity and Structure Analysis

**Figure 4:**
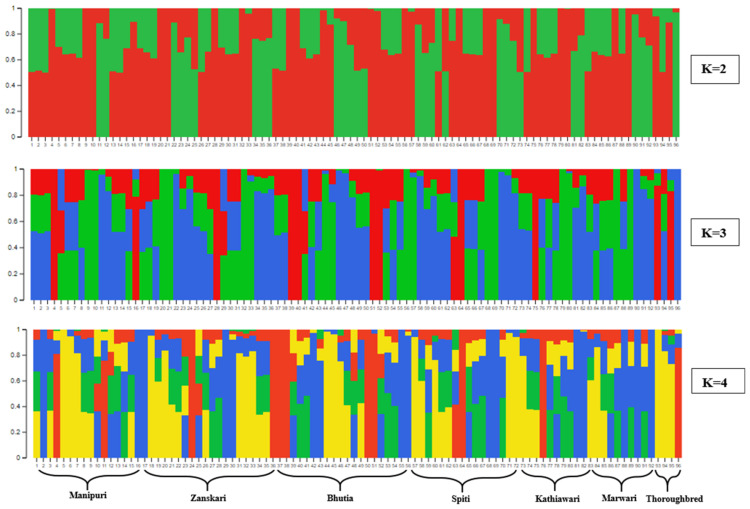
Population structure analysis of 96 individuals of *Equus caballus* based on SNPdata at K = 2, 3 and 4 presented by different colors inferred by Structure analysis. Each bar represents an individual, where different colors in that individual represent the estimated admixture using the Q statistic.

To comprehend the pattern of the genetic structure, a Bayesian clustering analysis was conducted using STRUCTURE. This population structure analysis was conducted with K values ranging from 2 to 7 with an admixture model and five iterative runs for each K value. An Evanno test was further conducted to determine the log-likelihood (LnP(D)) values and ΔK between for each K number. A population structure analysis of all 96 individuals from different breeds of *Equus caballus* based on SNPdata at K = 2, 3 and 4 is represented in Figure 4. Here, each bar represents an individual, where different colors for that particular individual show the estimated admixture from the ancestral population, calculated using the Q statistic. At K = 2, 3 and 4, all seven breeds with 96 samples were divided into two, three and four subpopulations, respectively (Figure 4), indicating the inferred ancestral population. The structure analysis at K = 2 suggested that admixture has occurred to a greater extent, as pure lines were present in a lesser amount, and this was also validated with the help of PCA results. A similar admixture pattern was detected with K = 3 and k = 4 as clustering criteria, and again this was confirmed with PCA results. It was observed that most of the population exhibited a certain amount of admixture and contained far less pure lines. Bhutia had the least diversity, followed by Marwari, compared to other breeds which were under study. As a whole, it can be inferred that high levels of introgression have ensued to a wider extent amongst the breeds under study.

### 3.4. Gene Annotation and Enrichment Analysis

**Figure 5:**
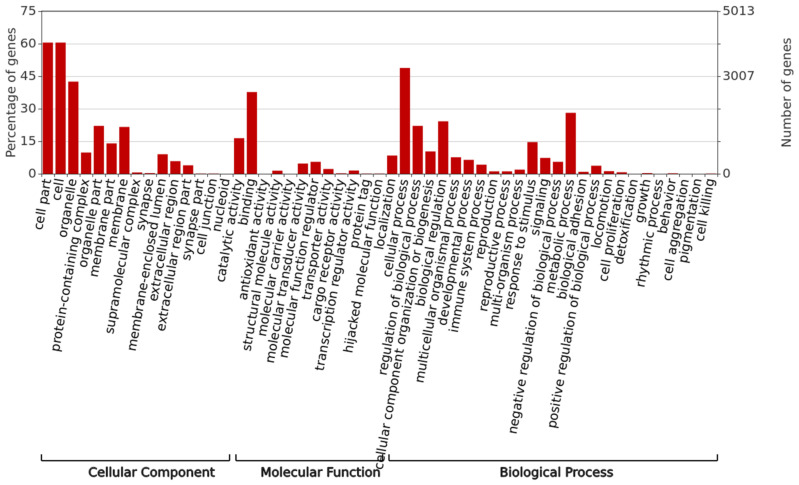
Gene function with their counts according to the category in which they fall.

SNP annotation by SnpEff produced total of 18,395 candidate genes within ROH islands. In all the breeds, GO terms and pathways associated with these genes were verified for evidence of functional enrichment. This produced 6757 genes which were further categorized into Biological Processes, Molecular Functions, and Cellular Components. The details of their GO IDs have been provided in Supplementary Sheet S2. Detailed gene ontology segregation according to the category has been provided in Figure 5.

Pathway analysis revealed that most of the genes were involved in metabolic pathways, followed by pathways related to cancer. Most of the genes covered under ROH islands were related to various diseases in horses, directly or indirectly. Top genes based on gene count and *p*-value were chosen and plotted. Details of all the pathways along with their *p*-values and gene count have been provided in Supplementary Sheet S3, Supplementary Figure S1.

### 3.5. QTL Analysis

It was observed that the maximum number of QTLs (52) was present in Zanskari, while Manipuri possessed the minimum number of QTLs, i.e., 42. Details based on class have been provided in Table 3. Maximum QTLs which were involved were linked with Wither’s height, which is the sub-category of the Growth trait in all seven breeds. Another important QTL that was identified was CMO: a body movement measurement which determines the type of gait in the horse. (This trait falls under the performance category of traits). Other traits which were mined during QTL analysis are insect bite hypersensitivity, racing ability, osteochondrosis, semen quality, and jumping ability. Details of all the traits are provided in Supplementary Sheet S4. Various important genes associated with different traits have been provided in Table 4.

### 3.6. Development of Equine SNP Marker Database

In the Equine SNP marker database (EqSNPDb), ROHs have been catalogued for all seven equine breeds along with all the samples in the form of a database. The ROH page provides an interactive interface where the user can choose the breed name as well as the chromosome number to retrieve the information of a particular breed. The result table provides the sample names corresponding to that breed, chromosome number, SNP ID, SNP position, length of ROH, and the number of SNPs in that ROH. SNPs page provides the genotype details of all the 620,721 SNPs in a tabular form. EqSNPDb is freely accessible at http://webtom.cabgrid.res.in/eqsnpdb/.

## 4. Discussion

In this study, Axiom™ Equine Genotyping Array Chip was used to identify the ROH islands in seven Indian horse (*Equus caballus*) breeds. According to the study published by Willing et al., Colli et al., and Utsunomiya et al. [27,28,29], a sample size can be as small as 4–6 and the polymorphic SNPs markers under investigation must be sufficiently large. To obtain better results, we have tried to fulfill both conditions by taking sample size accordingly for all the seven breeds, and the 620,721 variants were used for the ROH study. Amongst 5444 ROH islands, the mean number of ROH per animal was highest in Spiti and lowest in Bhutia. The maximum number of ROHs was present in the Zanskari breed, followed by Spiti distributed over all 31 chromosomes, and Thoroughbred contained least ROHs. ROH islands give a direct breed signature, as reported in the study of Ovine SNP 50 K in which 17 unique ROH islands could segregate 13 breeds under study [30]. ROH islands can also provide information about the genes associated with the desired traits and contribute to the inbreeding estimation of synthetic breed population, i.e., artificial selection [31].

The maximum amount of genome coverage was shown by the Spiti breed, which was in coherence with a large number of ROHs, while the minimum coverage was shown by the Manipuri breed. The length of ROH has a negative correlation with the time of co-ancestry because the random recombination events interrupt the transfer of lengthy chromosomal segments in the long run [32,33]. Thus, long ROH (>8 Mb) in Zanskari must have arrived due to inbreeding of up to five generations. On the contrary, the genomic segments possessing short ROHs (~1–8 Mb) have a greater relatedness, historically [34].

Among the seven breeds, four of them (Bhutia, Spiti, Kathiawari, Thoroughbred) had a greater portion of their autosome genome covered by ROH. The genomic inbreeding coefficient was low (FROH > 1 Mb) in all the breeds except Thoroughbred. These estimates of inbreeding are in agreement with the abundance and length of ROH in all the samples.

Upon gene enrichment analysis, a total of 6757 genes were annotated as functional. Upon analysis, it was revealed that the maximum number of genes was a part that was responsible for cellular components. Some of the important genes that were identified are from the AGPAT1, CLEC4, and CFAP20 families [35,36,37] and are involved in lipid biosynthesis, part of the C-type lectin domain that contains and regulates cell motility. Many of the genes like OAS1 and BPIF that are involved in immune response were also located in the study [38]. CRK-like proto-oncogene (CRKL) was also mined, and this plays a vital role in the regulation of immune cell functions that lead to cell migration and adhesion, cell proliferation, differentiation, and finally apoptosis, as suggested in a study involving humans [39]. ROH can act as a selection signature over the genome of a population to indicate the genes associated with significant traits leading to differentiation of the breed. Such studies have been previously carried out in sheep to study the role of genes lying near ROHs to identify the important traits like growth, body weight, meat quality/production, wool production and pigmentation, etc. [40].

ROH can also contribute to the conservation program of the breed by keeping in mind the level of inbreeding, pedigree comparison and heterozygosity correlation, followed by the construction of breed specific model for conservation rather than a generalized model for breed conservation. It can be used for analysis of animal admixture and monitoring of effective population size rather than census size for effective conservation programs [41]. Besides, the STRUCTURE analysis result gives the idea of introgression of alleles (i.e., breed admixture/ dilution) and the historical migration of individuals among the populations as well as common ancestry, if any, for different numbers of clusters [42].

It has been reported that FROH provides insight for inferring details about the history as well as about inbreeding levels of a population. Therefore, ROH has been used for the conservation of endangered populations for which genealogical information is not available [43].

In our study, while considering QTL annotation, maximum QTLs were linked with Wither’s height trait ontology that falls under the growth trait in all seven breeds. Various other traits that were present included insect bite hypersensitivity, racing ability, osteochondrosis, semen quality, and jumping ability. ROH patterns can reveal the genomic regions containing alleles associated with the traits under selection, and hence can predict QTL. Thus, they provide insight into genes related to a variety of traits, as reported in sheep [44]. Additionally, ROH association studies have been found to be more effective for explaining genetic variance concerning SNP [45].

## 5. Conclusions

Our study highlights the characterization of autozygosity in seven important horse breeds of India, with the main focus of short (ROH > 1 Mb) and long (ROH > 8 Mb) ROH regions. By monitoring genomic inbreeding (FROH), it was revealed that most of the breeds are mostly random bred and hence preserve sufficient genetic variability. The main genes underlying these ROH regions were mainly responsible for various metabolic functions, constituted cell parts, or were a part of major biological processes.

## Figures and Tables

**Table 1:** Genomic distributions and descriptive statistics of ROH in different horse breeds.

Breeds	n_ROH_	Range ROH	NM_ROH_	MGL_ROH_ (Mb)	MGP_ROH_ (%)	AL_ROH_ (Mb)	F_ROH_ > 1 Mb	F_ROH_ > 8 Mb	F_HOM_	r (F_ROH_ > 1 Mb—F_HOM_)	r (F_ROH_ > 8 Mb—F_HOM_)
Manipuri	863	13–127	1168.62	242.88	9	5.1	0.247	0.266	0.542	0.295	0.277
Zanskari	1169	17–133	1100.94	295.5	10.94	4.89	0.285	0.189	0.546	0.261	0.357
Bhutia	961	20–116	1212.11	301.35	11.16	5.28	0.238	0.202	0.541	0.303	0.339
Spiti	1083	26–154	1139.23	409.4	15.16	5.13	0.288	0.2	0.553	0.265	0.353
Kathiawari	630	18–147	1286.31	330.32	12.23	5.5	0.28	0.283	0.561	0.281	0.278
Marwari	574	22–111	1018.45	268.7	9.95	4.65	0.284	0.219	0.545	0.261	0.326
Thoroughbred	164	18–114	1350.84	346.63	12.84	5.71	0.214	0.37	0.555	0.341	0.185

n_ROH_: Total number of ROH per breed; NM_ROH_: mean number of ROH in a breed; MGL_ROH_: breed-wise mean genome length covered by ROH in Mb; MGP_ROH_: breed-wise mean genome proportion covered by ROH in percentage; AL_ROH_: average length of ROH (Mb) in a breed; F_ROH_: inbreeding coefficient based on ROH; F_HOM_: inbreeding coefficient based on homozygous loci; r: Spearman’s correlation coefficient.

**Table 2:** Statistics of ROH observed in diverse Indian horse breeds under different length classes (ROH1–2 Mb, ROH2–4 Mb, ROH4–8 Mb, ROH8–16 Mb, ROH > 16 Mb).

Breeds	Manipuri	Zanskari	Bhutia	Spiti	Kathiawari	Marwari	Thoroughbred
n_ROH_	863	1169	961	1083	630	574	164
ROH 1–2 Mb	430	576	501	517	277	299	77
N_ROH_ (percent)	49.83	49.27	52.13	47.74	43.97	52.09	46.95
Mean length (Mb) ± SD	1.36 ± 0.28	1.37 ± 0.28	1.34 ± 0.27	1.39 ± 0.28	1.39 ± 0.28	1.38 ± 0.27	1.35 ± 0.27
ROH 2–4 Mb	208	296	221	282	148	140	37
N_ROH_ (percent)	24.10	25.32	23.00	26.04	23.49	24.39	22.56
Mean length (Mb) ± SD	2.84 ± 0.60	2.79 ± 0.58	2.77 ± 0.57	2.78 ± 0.59	2.85 ± 0.61	2.77 ± 0.56	2.70 ± 0.52
ROH 4–8 Mb	101	167	113	161	105	73	19
N_ROH_ (percent)	11.70	14.29	11.76	14.87	16.67	12.72	11.59
Mean length (Mb) ± SD	5.52 ± 1.08	5.47 ± 1.10	5.71 ± 1.17	5.50 ± 1.03	5.58 ± 1.16	5.63 ± 1.03	5.38 ± 1.05
ROH 8–16 Mb	65	71	68	67	58	31	17
N_ROH_ (percent)	7.53	6.07	7.08	6.19	9.21	5.40	10.37
Mean length (Mb) ± SD	10.62 ± 1.97	11.20 ± 2.01	11.12 ± 2.34	10.73 ± 2.23	11.10 ± 2.32	10.72 ± 1.86	11.79 ± 2.27
ROH > 16 Mb	59	59	58	56	42	31	14
N_ROH_ (percent)	6.84	5.05	6.04	5.17	6.67	5.40	8.54
Mean length (Mb) ± SD	33.56 ± 22.86	40.68 ± 28.18	41.25 ± 30.11	43.80 ± 28.56	33.96 ± 19.20	36.32 ± 25.56	30.80 ± 17.85

**Table 3:** Number of traits based on classes in all the breeds.

Trait	Marwari	Bhutia	Spiti	Kathiawari	Zanskari	Manipuri	Thoroughbred
Health	13	15	13	16	16	13	17
Growth	12	18	15	15	17	14	18
Exterior	2	4	4	1	2	4	0
Reproduction	7	2	6	3	7	3	5
Performance	10	8	9	8	10	8	6
Total	44	47	47	43	52	42	46

**Table 4:** Genes associated with different traits.

Trait	Associated Genes
Health	FRZB, XIRP2, MATN1, COL1A2, ARHGAP26, TBC1D22A, CCNG1, GABRA6, TENM2, IGF1, ACAN
Growth	OXCT1, HMGA2, MYPN
Exterior	MGP, KIT
Reproduction	PLCZ1, NXPE3
Performance	SLC16A1, XIRP2, SOX5, CCT5, MRPL44, SERPINE2, CUL3, GIGYF2, INPP5D, MSTN, GRIN2B, SLC16A1, ARL15, DOCK8, HMGA2, DMRT3, MYPN, NXPE3, WDFY2, ATP7B, KCNRG, IGF1, CNTN3, PDZRN3, PPP4R2, GXYLT2

## Data Availability

All analysed data are available at http://webtom.cabgrid.res.in/eqsnpdb/. The same is mentioned in the manuscript also.

## Supplementary Materials

The following supporting information can be downloaded at: https://www.mdpi.com/article/10.3390/genes14081623/s1, Sheet S1: ROH variation chromosome wise for all breeds; Sheet S2: Gene ontology analysis results Sheet S3: List of important Pathways; Sheet S4: Detailed results of QTL analysis; Supplementary Figure S1. Pathway Analysis.
