# Supplementary figures and images for: Genome-Wide Single-Nucleotide Polymorphism-Based Genomic Diversity and Runs of Homozygosity for Selection Signatures in Equine Breeds
Source: Genes (Basel). 2023 Aug 14;14(8):1623. doi: 10.3390/genes14081623 (PMC10454598; doi:10.3390/genes14081623)

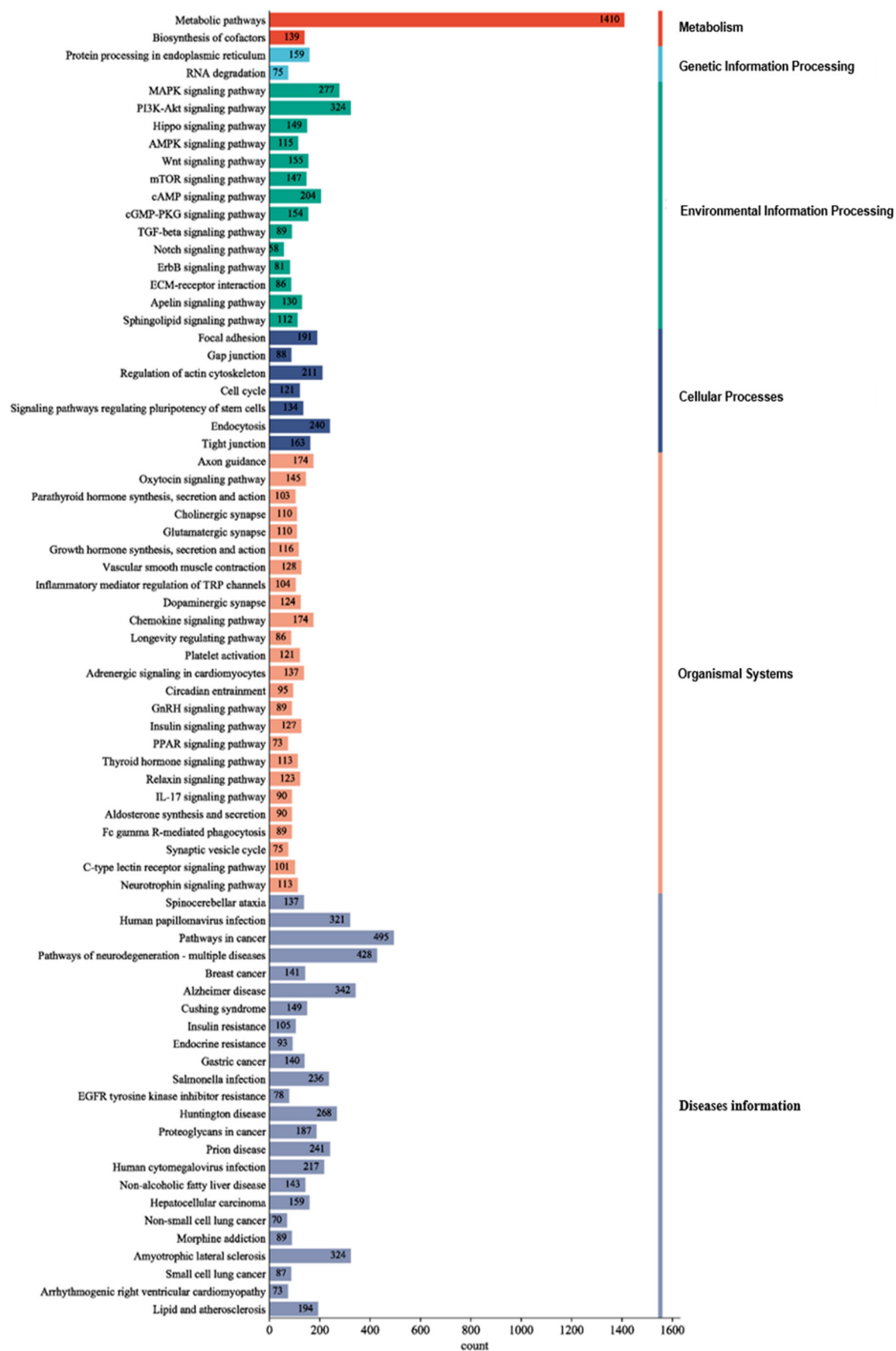

Supplement: Supplementary file 1 [file genes-14-01623-s001.zip › Figure S1.pdf]

## ROH variation chromosome wise for all breeds

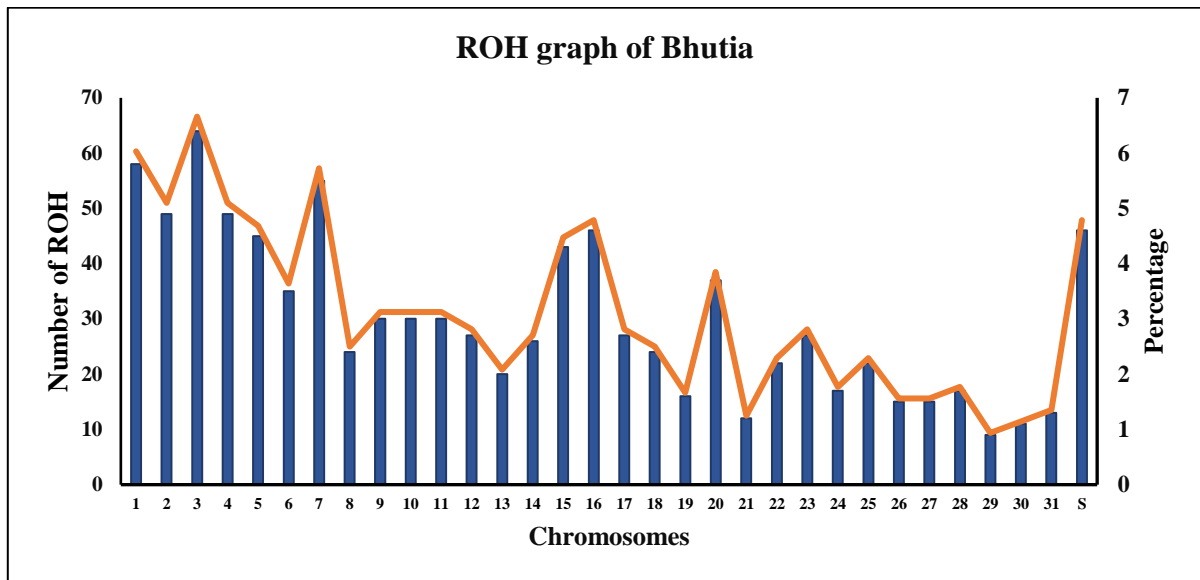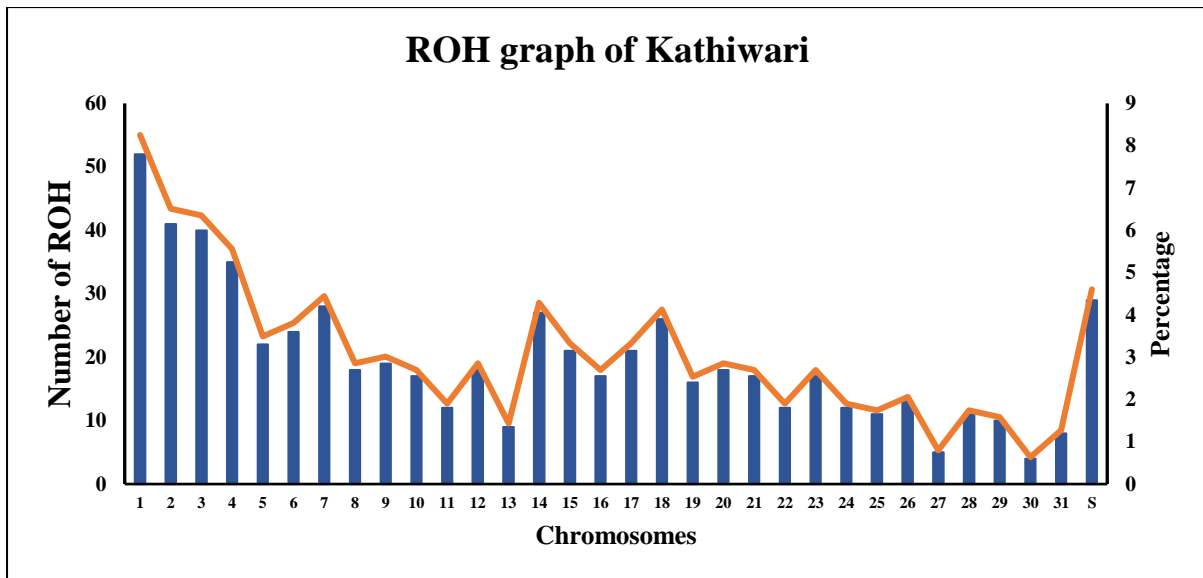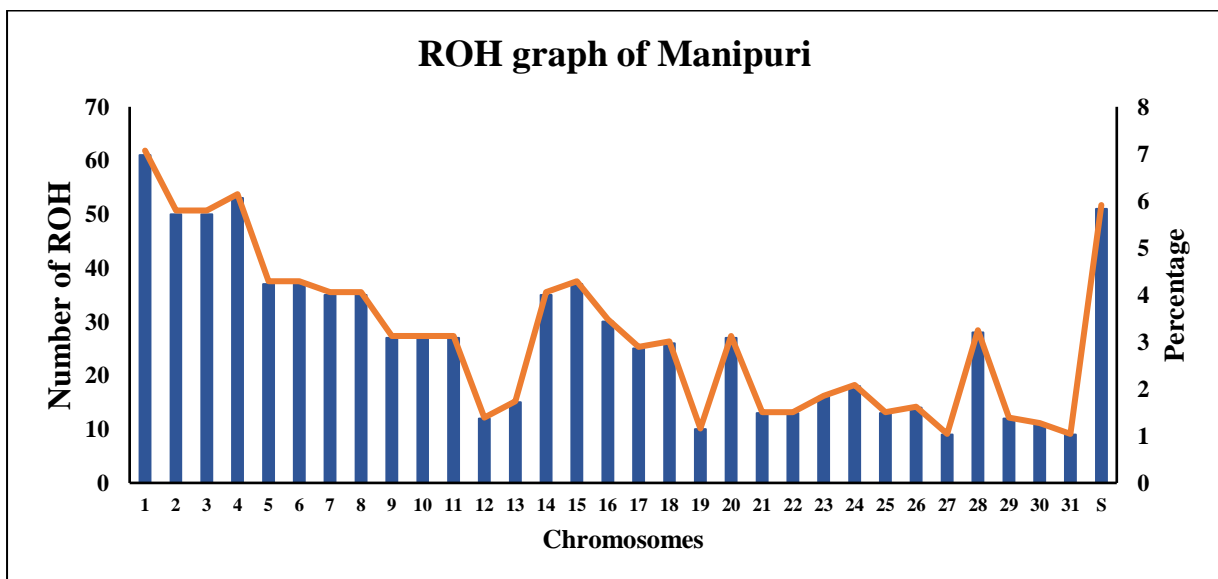

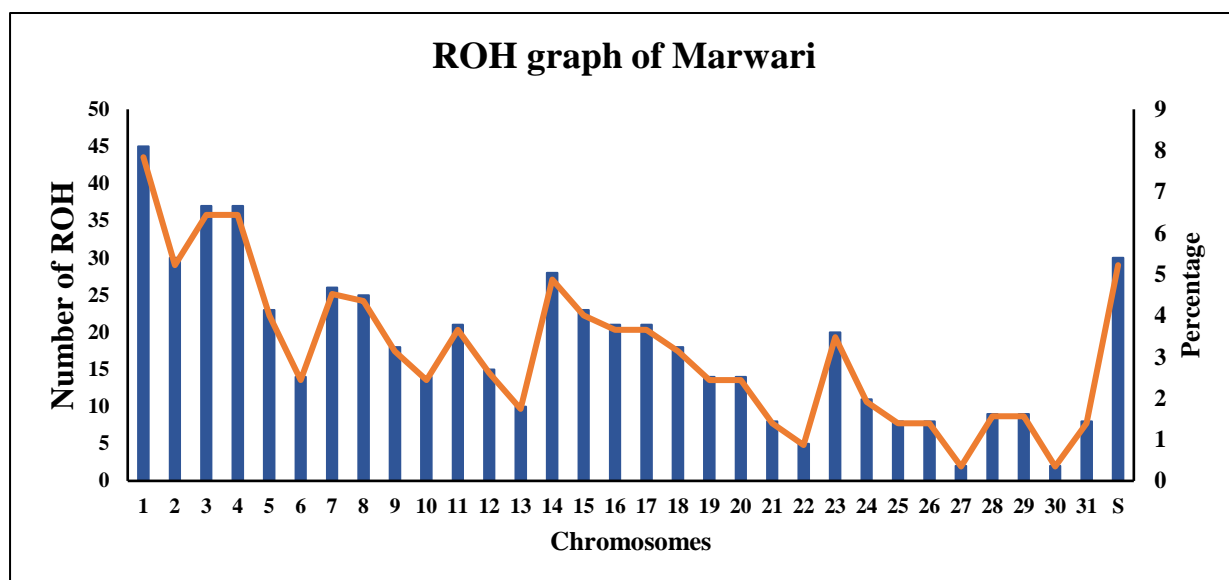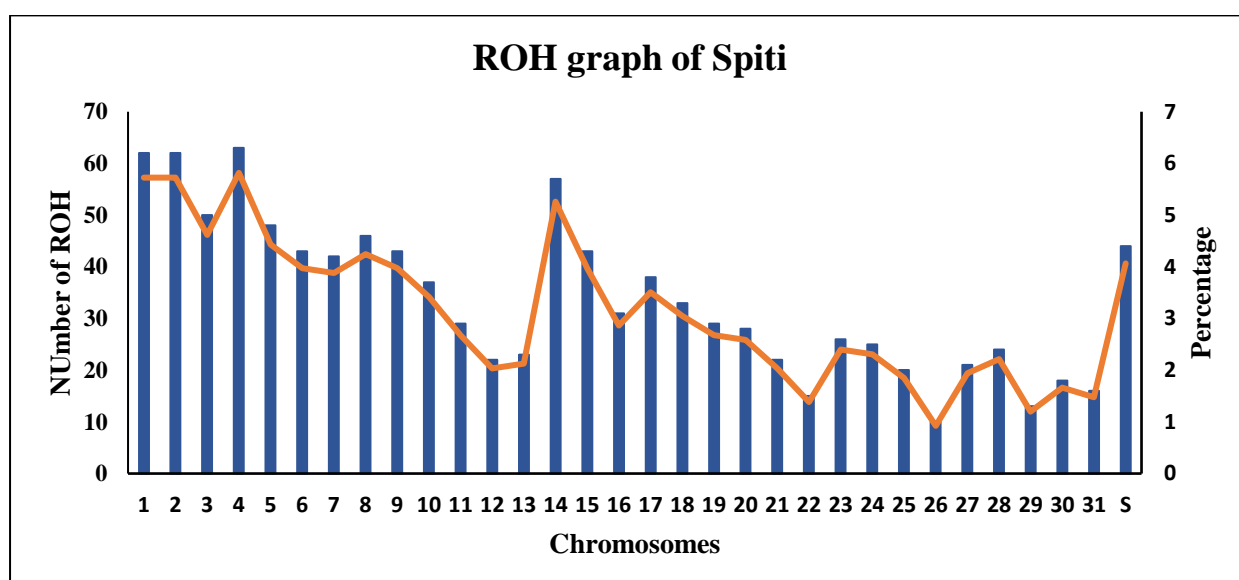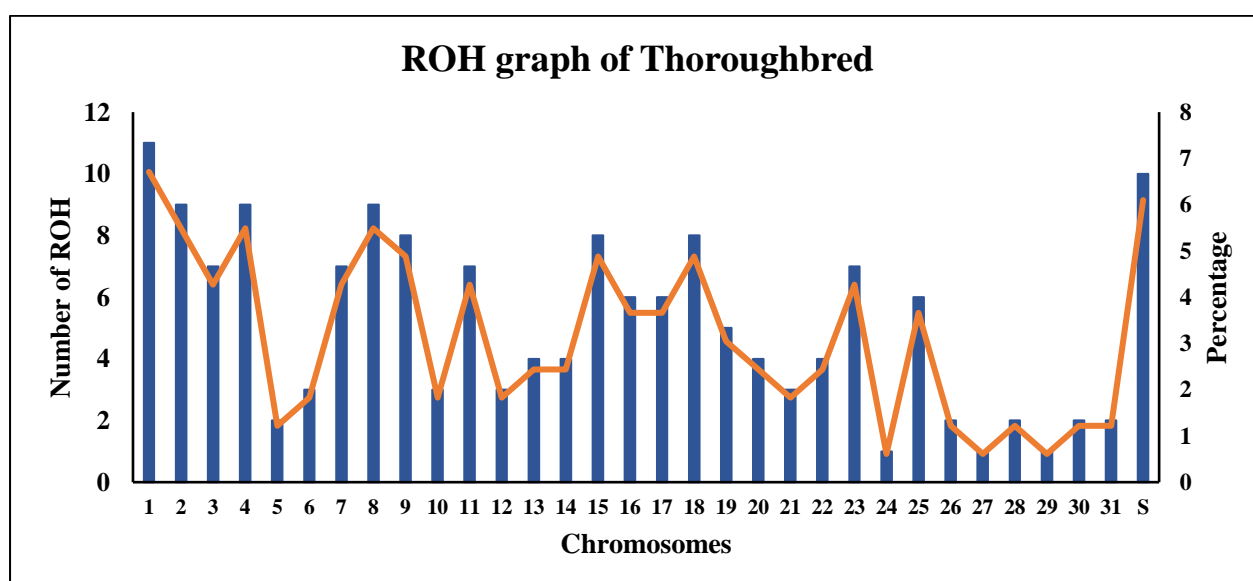

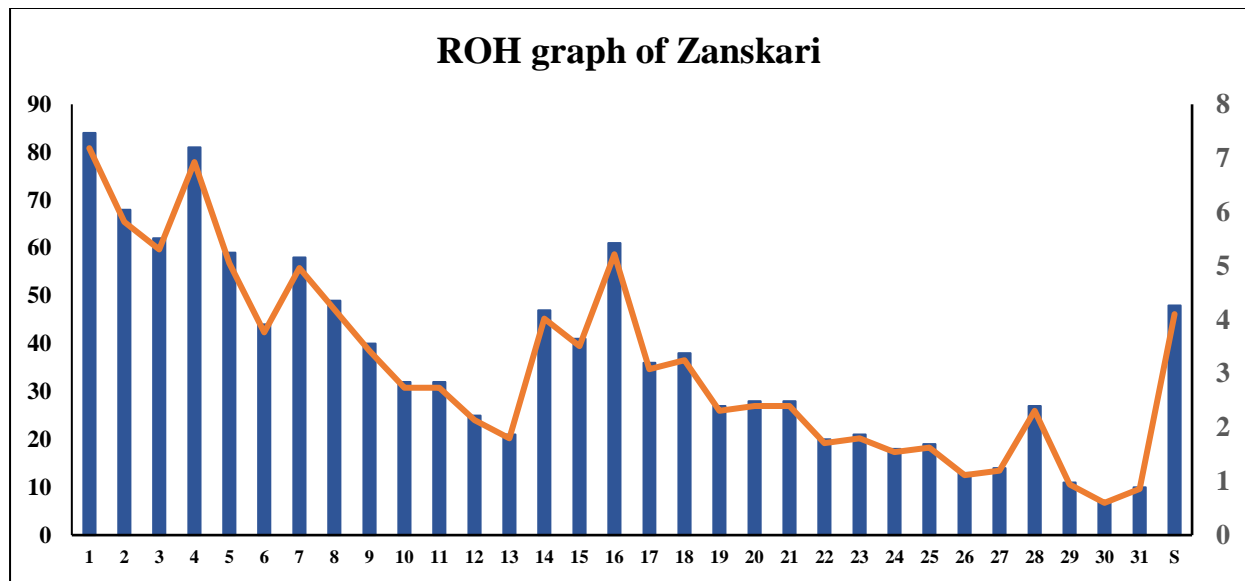

Supplement: Supplementary file 1 [file genes-14-01623-s001.zip › Supplementary_Sheet S1.pdf]
